# Psychotherapy Process Dynamics and Their Relation to Treatment Success Do Not Differ Across Diagnoses

**DOI:** 10.1002/cpp.70222

**Published:** 2026-01-17

**Authors:** Lennart Seizer, Leonhard Kratzer, Johanna Löchner, Helmut Schöller, Wolfgang Aichhorn, Günter Schiepek

**Affiliations:** ^1^ Department of Clinical Psychology and Psychotherapy for Children and Adolescents Friedrich‐Alexander‐Universität Erlangen‐Nürnberg Erlangen Germany; ^2^ Department of Child and Adolescent Psychiatry, Psychosomatics and Psychotherapy University Hospital of Tübingen Tübingen Germany; ^3^ German Center for Mental Health (DZPG) Tübingen Germany; ^4^ Department of Psychotraumatology Clinic St. Irmingard Prien am Chiemsee Germany; ^5^ Institute of Synergetics and Psychotherapy Research Paracelsus Medical University Salzburg Austria; ^6^ University Hospital of Psychiatry, Psychotherapy, and Psychosomatics, Paracelsus Medical University Salzburg Austria

**Keywords:** ambulatory assessment, diagnostic categories, dynamic pattern, ecological momentary assessment, monitoring, psychotherapy, therapy process questionnaire, transdiagnostic

## Abstract

Psychotherapy process research increasingly uses intensive longitudinal monitoring to capture dynamic patterns of change in patients. In this study, 283 psychiatric inpatients diagnosed with depression (*n* = 70), PTSD (*n* = 148), dissociative disorders (*n* = 26) or personality disorders (*n* = 39) completed the Therapy Process Questionnaire each evening over an average of 81.5 days per patient, yielding a total of 23,074 assessment days. We computed eight dynamic process characteristics, including variability, autocorrelation, instability and complexity, for each TPQ scale, both on average per patient and in a moving‐window approach. Then, the diagnostic groups were compared via one‐way ANOVAs with Bonferroni–Holm correction. No significant differences emerged across diagnoses in any average process characteristics or their change over time, indicating that idiosyncratic within‐person process dynamics overshadow diagnostic distinctions. Further, patients' clinical improvement was associated with rising mean levels and declining variability in positive emotions, mindfulness, insight and motivation. Again, these predictions of improvement were not moderated by patients' diagnoses. Our results support a transdiagnostic approach to measurement‐based care that leverages individual process characteristics to guide interventions, rather than relying on diagnostic categories to predict therapy trajectories.

## Summary

- Day‐to‐day psychotherapy process dynamics (e.g., variability, instability and complexity) do not differ meaningfully between inpatients with depression, PTSD, dissociative or personality disorders.
- Across all diagnoses, clinical improvement was associated with increasing mean levels and decreasing variability of positive emotions, mindfulness, insight and motivation for change.
- High‐frequency routine monitoring with the Therapy Process Questionnaire can be used in everyday practice to track both levels and fluctuations in these processes, providing early indications of whether a patient is moving toward recovery or remaining unstable.
- Since process–outcome relations did not differ across diagnostic groups, measurement‐based, process‐focused care and feedback systems may be implemented in a largely transdiagnostic way.

## 1 Introduction

Psychotherapy research has increasingly recognized the value of process monitoring to understand psychopathology and to inform treatment decisions. Routine outcome monitoring and feedback systems are increasingly being used in clinical practice (Moltu et al. 2025), with evidence that integrating regular patient self‐reports can improve therapy outcomes (Lambert et al. 2018). A key motivation for frequent assessments is to observe dynamic patterns that would be missed by coarse sampling, as psychological states and psychopathological symptoms can fluctuate markedly from day to day (Seizer et al. 2025; Schubert et al. 2025).

The theoretical rationale behind this intensive monitoring is grounded in dynamic systems theory and complexity science applied to human change processes. Over the past years, psychotherapy has increasingly been conceptualized as a nonlinear, dynamic and complex system (Hayes and Andrews 2020; G. K. Schiepek et al. 2017; Gelo and Salvatore 2016; G. Schiepek et al. 2014). Unlike a linear dose–response view, in which symptoms steadily improve in proportion to intervention over time, the dynamic systems perspective posits that psychological change trajectories often have complex shapes and discontinuities, corresponding to phase transitions in self‐organizing processes (G. Schiepek et al. 2020). Empirical studies of psychotherapy have indeed documented many nonlinear phenomena: abrupt sudden gains or losses in symptomatology (Lutz et al. 2013), rupture–repair oscillations in the therapeutic alliance (Gumz et al. 2012; Høgenhaug et al. 2024) or critical fluctuations as predictors of symptom changes (Olthof, Hasselman, Strunk, Aas, et al. 2020). This complexity is not merely random noise; it can be theoretically understood via concepts like attractor states (relatively stable patterns of thoughts/feelings) and bifurcations (switches between stability and instability as conditions change).

Importantly, these nonlinear dynamics do not only characterize symptom trajectories but also relational processes within therapy. For instance, Kratzer et al. (2024) demonstrated in a study of daily inpatient psychotherapy data that working alliance instability was significantly higher in patients with borderline personality disorder compared to those with major depressive disorder. Notably, greater dynamic complexity in the alliance predicted better outcomes in the patient group, suggesting that productive instability, likely reflecting adaptive rupture–repair cycles, may represent a core mechanism of change rather than a therapeutic deficit. This finding exemplifies how dynamic systems principles can be empirically observed in psychotherapy processes and underscores the importance of capturing temporal microdynamics rather than relying solely on static or averaged indicators.

Building on this perspective, previous research has examined whether dynamic profiles differ systematically across psychiatric diagnoses. Borderline personality disorder, for instance, is characterized clinically by mood and interpersonal instability, a fact partly reflected in quantitative diary studies: Borderline patients showed higher variability and instability in mood compared to depression patients (Trull et al. 2008) and bipolar patients (Tsanas et al. 2016) but not compared to PTSD and bulimia nervosa patients (Santangelo et al. 2014). Further, heightened inertia of shame has been found to be relatively specific to borderline patients, compared to depression and bipolar patients (Mneimne et al. 2018). In a comparison of participants with a lifetime history of Bipolar I or II, depression or anxiety disorder, greater variability and instability were observed for sad mood in the Bipolar II and depression groups. However, no group differences were observed for the inertia of sad or anxious moods (Lamers et al. 2018). Further, current depression/anxiety patients have been shown to exhibit higher instability of positive and negative affect compared to remitters and controls (Schoevers et al. 2021). In summary, while there are some reports on specific differences regarding dynamic pattern and diagnoses, these are not consistently found; and overall, literature on this topic is sparse.

In the present study, we investigated process characteristics in psychotherapeutic variables of psychiatric inpatients across four diagnostic groups—depression, personality disorder, dissociative disorder and PTSD—using daily self‐assessments. We focus on process characteristics derived from each patient's time series, like measures of variability, complexity and instability over time. Our aims were (1) to examine whether these process characteristics differ systematically by diagnostic categories, or whether the idiosyncratic patterns of each individual overshadow any diagnosis‐related trends, (2) how these process characteristics change over time during therapy, and (3) if changes in these process characteristics are related to treatment outcomes. These questions have both theoretical and practical significance. Theoretically, they test whether specific psychopathologies carry distinct ‘dynamic signatures’ or whether such distinctions fade when confronted with individual variability. Practically, understanding these patterns and their relation to diagnoses can inform the development of monitoring systems and approaches to personalized treatment.

## 2 Methods

### 2.1 Participants

The sample consists of 283 patients who were treated in an inpatient psychotherapy setting between 2017 and 2022 at two clinics in Germany and Austria. The patients who were included from the University Hospital of Psychiatry, Psychotherapy and Psychosomatics (Salzburg, Austria) were treated either at the day‐treatment centre for psychotraumatology or at the inpatient department of this hospital. The treatment programme is integrative including skills training, psychoeducation, mindfulness‐based stress reduction, schema therapy, therapy groups, movement/sports and ergo‐therapy and client‐focused psychotherapy. Patients with PTSD and dissociative disorder underwent stepwise trauma confrontation, EMDR and the therapy concept of Nijenhuis and colleagues (van der Hart et al. 2006). The department of Psychotraumatology at Clinic St. Irmingard (Germany) utilizes a personalized, multicomponent inpatient treatment model integrating skills training with trauma‐focused individual therapy (Kratzer et al. 2023). All patients could be assigned to one of the following diagnostic groups based on their primary diagnosis: depression disorder (*n* = 70), PTSD (*n* = 148), dissociative disorder (*n* = 26) and personality disorder (*n* = 39). During their inpatient stay, the patients completed a daily questionnaire each evening. The average measurement period (time series length) was 81.53 days (SD = 34.67; range = 50 to 477), and each patient had less than 5% of missing assessments, resulting in a total of 23,074 completed measurement days. There were no significant differences in time series length between the diagnostic groups.

### 2.2 Therapy Process Questionnaire

The Therapy Process Questionnaire (TPQ) is a self‐rating questionnaire developed for daily monitoring of psychotherapeutic processes (G. Schiepek et al. 2019). The TPQ comprises 43 items that are aggregated into seven subscales: ‘well‐being and positive emotions’ (WPE), ‘relationship with fellow patients’ (RFP), ‘therapeutic relationship and clinical setting’ (TAS), ‘emotional and problem intensity’ (EPI), ‘insight/confidence/therapeutic progress’ (ICP), ‘motivation for change’ (MOT) and ‘mindfulness/self‐care’ (MSC). The internal consistency of the scales is high (Cronbach's *α* ranging from 0.87 to 0.94). The patients completed the questionnaire each evening on their mobile phones using the Synergetic Navigation System (G. Schiepek, Aichhorn, et al. 2016).

### 2.3 Treatment Outcome

Treatment outcome was evaluated using the problem intensity subscale from the TPQ. For each participant, a baseline score was calculated as the mean during the first week of therapy, and an end‐of‐treatment score was calculated as the mean of the final treatment week. Averaging across an entire week, rather than relying on single‐day values, provided a more stable estimate of patients' typical levels of perceived problem severity (Seizer et al. 2025). Further, this scale captures individuals' subjective perception of how problematic their experiences are, rather than focusing solely on symptom counts, which makes it appropriate for a diagnostically diverse clinical population (see for a similar approach Olthof, Hasselman, Strunk, van Rooij, et al. 2020). On average, problem intensity decreased by 7.49 (SD = 20.12). Patients were classified according to the Reliable Change Index (RCI) (Jacobson and Truax 1991). This method tests whether a participant's change score exceeds what could be expected from measurement error, taking into account the reliability of the assessment tool. Based on the RCI formula, the final sample consisted of 67 reliably improved and 216 non‐improved patients.

### 2.4 Data Analysis

All statistical analyses were performed in *R* 4.4. First, for each patient's time series several dynamic process characteristics were calculated, which are listed in Table 1. Subsequently, these were compared across the diagnostic groups using one‐way ANOVAs. The Bonferroni–Holm method was applied as a correction for multiple testing. In a second step, the process characteristics were computed in moving windows of 2 weeks for each patient. To summarize temporal changes over the therapy period across all patients, we regressed each process characteristic on time in mixed‐effects models with full random effects. Further, differences in the slope of time across diagnostic groups were tested using one‐way ANOVAs. In a third step, to test whether within‐patient changes in process characteristics predicted improvement, we fit logistic regressions with reliable improver status as the dependent variable and the slope of time as the predictor. Further, we tested whether this association between trends in process characteristics and improvement varied by diagnostic group by including a moderation term to the logistic regressions. Subsequently, a likelihood‐ratio test was run to evaluate whether the moderation model significantly improved fit to the data. Again, the Bonferroni–Holm correction was applied. Note that the logistic regressions were run with all TPQ scales except emotional and problem intensity as predictors, as a subset of items from this scale was used to compute the treatment outcome.

**TABLE 1:** Description of the dynamic metrics calculated from the patients' time series.

Metric	Explanation
ACF	Autocorrelation function: Pearson correlation between each value and the next value in the time series; reflects temporal inertia
DC	Dynamic complexity: Product of a fluctuation measure (amplitude and frequency) and a distribution measure, computed within moving 7‐day windows and then averaged
Mean	Arithmetic mean: Average of all measurements for a given variable
PAC	Percentage of abnormal changes: Fraction of successive differences whose absolute magnitude exceeds 2× SD; indicates instability or abrupt changes
Rel. SD	Relative standard deviation: Standard deviation divided by the mean, expressed as a percentage; reflects variability relative to the level
RMSSD	Root mean square of successive differences: Square root of the mean of squared differences between consecutive measurements; quantifies short‐term variability
SD	Standard deviation: Square root of the mean of squared deviations from the mean; reflects overall variability
Entropy	Shannon entropy: Measure of unpredictability in the data; higher values indicate more randomness and lower values greater predictability.

## 3 Results

### 3.1 Average Process Characteristics

**FIGURE 1:**
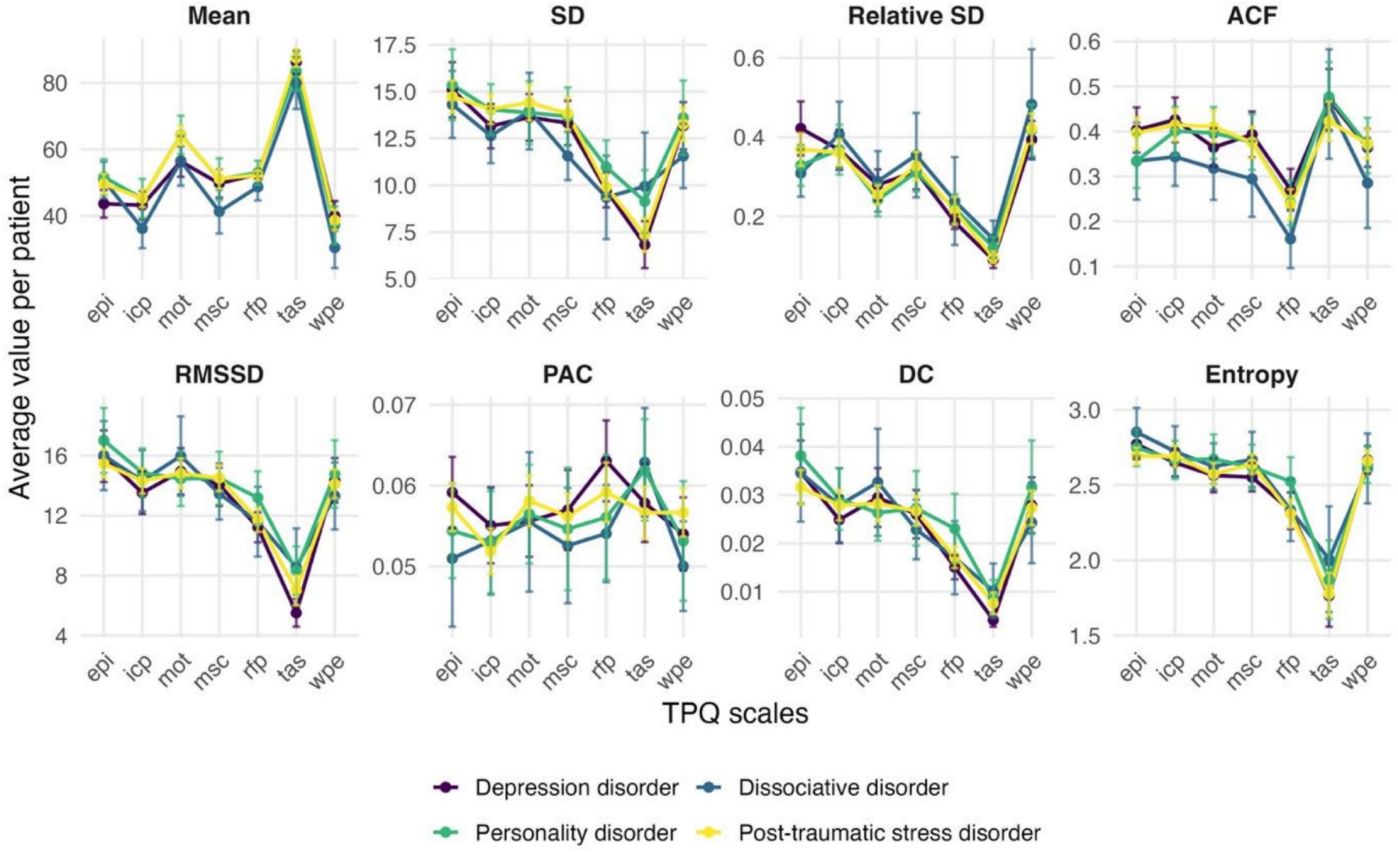
Comparison between the diagnostic groups in the process characteristics of the Therapy Process Questionnaire (TPQ) scales: epi: ‘emotional and problem intensity’, icp: ‘insight/confidence/therapeutic progress’, mot: ‘motivation for change’, msc: ‘mindfulness/self‐care’, rfp: ‘relationship with fellow patients’, tas: ‘therapeutic relationship and clinical setting’, wpe: ‘well‐being and positive emotions’. The average values with 95% confidence intervals are shown. The metrics are described in Table 1.

The average process characteristics were calculated for each TPQ scale per patient over their whole time series. We found no significant differences between the diagnostic groups in these average process characteristics per patient in any TPQ scale. Summary statistics on these process characteristics are available in Data S1 and detailed results of all group comparisons in Data S2. Figure 1 shows a comparison of the TPQ scales' average process characteristics across patients with different diagnoses.

### 3.2 Changes Over Time in Process Characteristics

**FIGURE 2:**
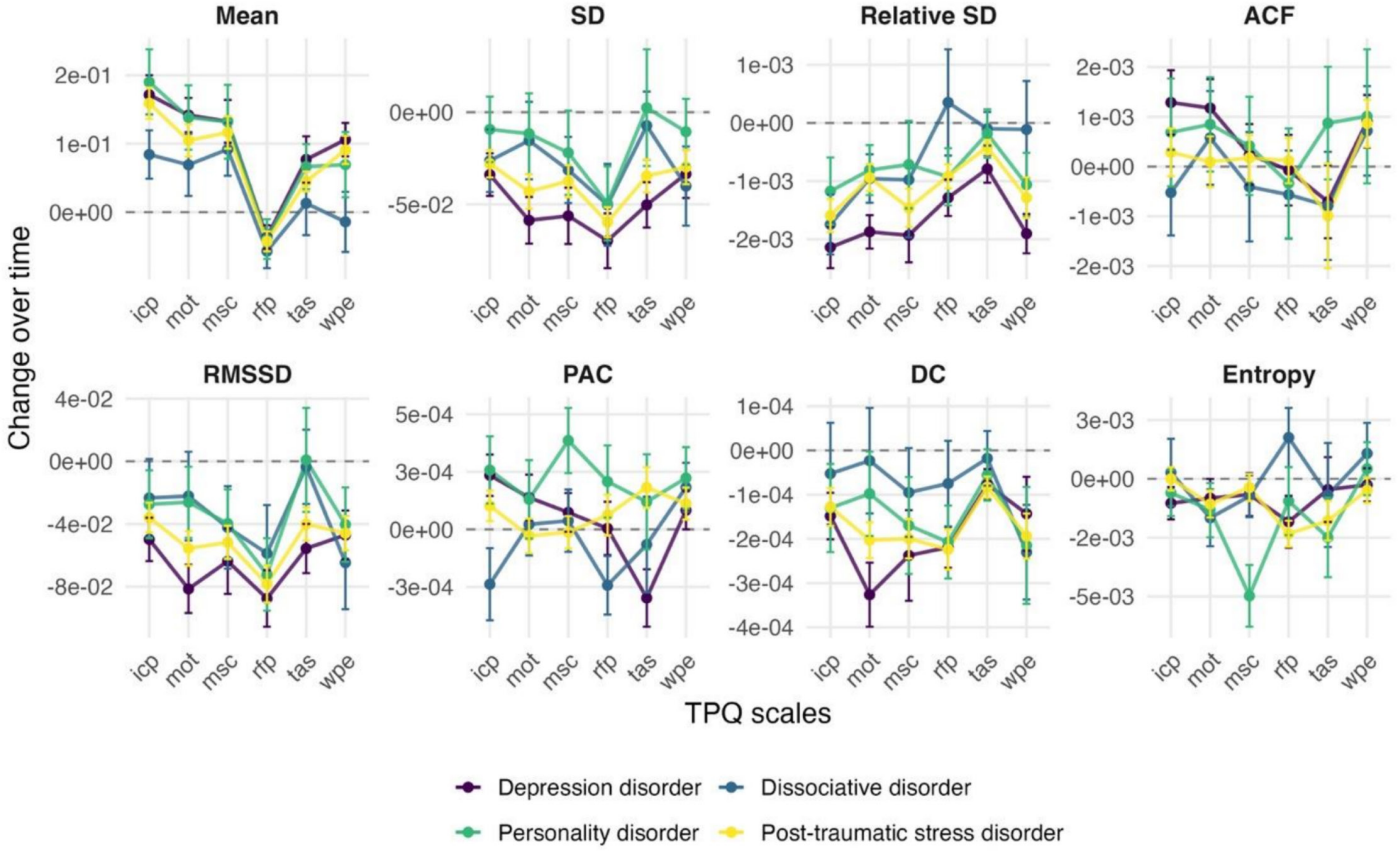
Changes over time in the process characteristics calculated in 14‐day moving windows. The change is given as the regression coefficient of time with 95% confidence intervals. Epi: ‘emotional and problem intensity’, icp: ‘insight/confidence/therapeutic progress’, mot: ‘motivation for change’, msc: ‘mindfulness/self‐care’, rfp: ‘relationship with fellow patients’, tas: ‘therapeutic relationship and clinical setting’, wpe: ‘well‐being and positive emotions’. The dynamic metrics are described in Table 1.

To investigate how the process characteristics of each patient changed during therapy, they were calculated in 14‐day moving windows for each TPQ scale. In a mixed‐effects model, the resulting time series of process characteristics were regressed on time to estimate the average changes across all patients. The mean of most TPQ scales increased over time but decreased for emotional and problem intensity and relationship with fellow patients. The SD, RMSSD and DC significantly decreased over time for all scales. The relative SD decreased for all scales except for emotional and problem intensity, for which it increased. Entropy decreased for therapeutic relationship, mindfulness/self‐care, motivation for change and relationship with fellow patients. The ACF increased for emotional and problem intensity and positive emotions. The PAC increased for positive emotions and insight/confidence/therapeutic progress. Further, the changes over time were estimated for each individual patient to compare them across diagnostic groups. This comparison across groups is illustrated in Figure 2. There were no significant differences in the changes of process characteristics over time. Detailed results on the changes over time are available in Data S3 and on the group comparisons in Data S4.

### 3.3 Changes Over Time in Process Characteristics Predicting Clinical Improvement

**FIGURE 3:**
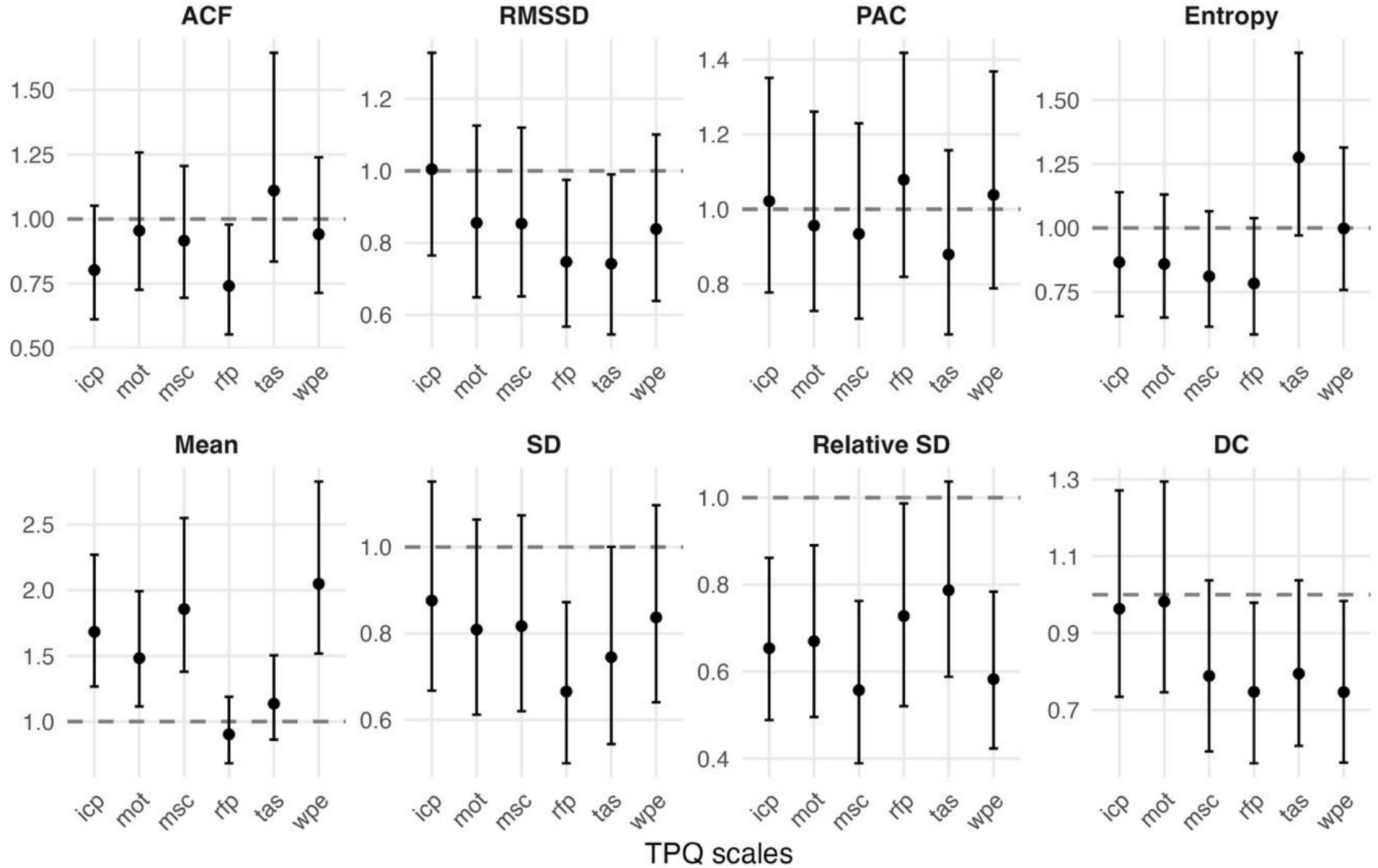
Odds ratios with 95% confidence intervals of how linear change over time in the process characteristics of the Therapy Process Questionnaire (TPQ) scales are related to improvement. Odds ratio values over 1 indicate an increase over time is associated with more probable improvement. Epi: ‘emotional and problem intensity’, icp: ‘insight/confidence/therapeutic progress’, mot: ‘motivation for change’, msc: ‘mindfulness/self‐care’, rfp: ‘relationship with fellow patients’, tas: ‘therapeutic relationship and clinical setting’, wpe: ‘well‐being and positive emotions’. The dynamic metrics are described in Table 1.

Logistic models were estimated to test the relations of changes in process characteristics over time (the trend slopes from 14‐point moving windows) to reliable improvement (Figure 3). Patients with increasing means of positive emotions (OR = 2.05, *p* < 0.001), mindfulness/self‐care (OR = 1.86, *p* = 0.002), insight/confidence/therapeutic progress (OR = 1.68, *p* = 0.006) and motivation for change (OR = 1.48, *p* = 0.040) over the therapy period had a higher probability of reliable improvement. Similarly, patients with decreasing relative SD of positive emotions (OR = 0.58, *p* = 0.006), mindfulness/self‐care (OR = 0.56, *p* = 0.006), insight/confidence/therapeutic progress (OR = 0.65, *p* = 0.025) and motivation for change (OR = 0.67, *p* = 0.040) also had a higher probability of reliable improvement. To test for differences in these predictive relations between diagnostic groups, likelihood‐ratio tests were run comparing models with and without an interaction of time and diagnosis. These yielded no significant moderating effects. Thus, the relationship between changes in process characteristics and improvement did not differ reliably across diagnostic groups. More detailed information on the logistic regressions is available in Data S5 and Data S6.

## 4 Discussion

In this study, we have investigated characteristics of patients' psychotherapy process characteristics and how these vary over time and between diagnostic groups. We found no significant differences in the average psychotherapy process dynamics per patient across depression, personality disorder, PTSD and dissociative disorder. Further, we found no differences between these groups in how the characteristics change over time. These null results suggest that dynamic process features may represent idiosyncratic patterns of each individual rather than any diagnosis‐related patterns. However, systematic differences emerged between the TPQ scales. For example, across all patients, therapeutic alliance had the highest mean and inertia and the lowest variability, instability and complexity. Moreover, increases over time in the mean and decreases in the variability of positive emotions, mindfulness/self‐care, insight/confidence/therapeutic progress and motivation for change were associated with clinical improvement.

To our knowledge, this is the first study to compare the dynamics of therapy process variables between different diagnostic groups. Our results mirror diary‐ and EMA‐based research on everyday emotions, showing that instability is elevated in various clinical groups but does not reliably distinguish among them. Santangelo et al. (2014) found that borderline, PTSD and bulimia nervosa patients all exhibited high moment‐to‐moment mood fluctuations without clear group differences. Further studies documented heightened mood variability in borderline versus depressive or bipolar samples only under certain conditions but also noted that some comparisons (e.g., versus PTSD) yielded no differences (Tsanas et al. 2016; Trull et al. 2008). Collectively, these studies on affect dynamics and our data on psychotherapy processes underscore that within‐person dynamics often transcend diagnostic boundaries, supporting a shift toward transdiagnostic process‐based models of psychopathology (G. K. Schiepek et al. 2017).

The changes over time associated with clinical improvement were marked by rising mean levels and declining variability in positive emotions, mindfulness, insight and motivation. Such patterns may indicate improved emotion regulation and greater integration of therapeutic gains over time (de Felice et al. 2021). These results fit with findings of previous intensive longitudinal studies, where lower levels in several psychopathology symptoms were associated with less variable affect dynamics (Schoevers et al. 2021; Houben et al. 2015). Increases in mindfulness and self‐care may signal improvements in emotional and behavioural regulation, consistent with evidence that mindfulness‐based interventions can successfully be used in the treatment of mental health disorders (Goldberg et al. 2022; Keng et al. 2011). Likewise, increasing insight into one's own problems and motivation for change has been identified as key mediators of change across therapeutic modalities (Jennissen et al. 2018; Antony et al. 2005). The concurrent decrease in variability may thus signify a consolidation phase in the therapeutic process, as patients move from emotional instability and motivational ambivalence toward more stable adaptive functioning. This pattern resonates with dynamic systems perspectives that conceptualize improvement as the emergence of new attractor states after periods of instability (Hayes and Andrews 2020; G. Schiepek et al. 2020).

Taken together, these results highlight that both the direction (mean increase) and the form (reduced variability) of change provide meaningful information about recovery processes, underscoring the value of monitoring not only average symptom levels but also temporal fluctuations in psychotherapy processes. And further, that such measurement‐ and process‐based care may be applied across diagnostic groups, e.g., to flag meaningful fluctuations across all patient groups, as we found no significant differences in how they relate to treatment outcomes across diagnoses (Hofmann and Hayes 2019; G. Schiepek, Stöger‐Schmidinger, et al. 2016). In the future, feedback from such monitoring applications may help clinicians to time process‐sensitive interventions (Seizer et al. 2024; Löchner et al. 2024). Moreover, since dynamic markers like temporary instability may signal opportunities for therapeutic reorganization, clinicians could use them to guide treatment decisions in a precision‐health framework, tailoring interventions to each individual's unique temporal profile.

## Funding

The authors have nothing to report.

## Ethics Statement

The study has been approved by the Ethics commission of Salzburg County Governance (415‐E/1068/3‐2009).

## Conflicts of Interest

The authors declare no conflicts of interest.

## Supporting information


**Data S1:** Supporting information.

## Data Availability

The data that support the findings of this study are available from the corresponding author upon reasonable request.
