# Supplementary material for: Psychotherapy Process Dynamics and Their Relation to Treatment Success Do Not Differ Across Diagnoses
Source: Clin Psychol Psychother. 2026 Jan 17;33(1):e70222. doi: 10.1002/cpp.70222 (PMC12811794; doi:10.1002/cpp.70222)
Supplement: Supplementary file 1 — Data S1: Supporting information. [file CPP-33-e70222-s001.html]

Supplement


# Supplement

### **S1** Summary statistics of process characteristics

Mean (SD) of process characteristics by TPQ scale, calculated
over the entire time series for each patient.


| TPQ scale | mean | sd | rel\_sd | acf1 | rmssd | pac | dc | shan |
| --- | --- | --- | --- | --- | --- | --- | --- | --- |
| epi | 48.55 (17.7) | 14.87 (5.47) | 0.37 (0.25) | 0.38 (0.21) | 15.86 (6.33) | 0.06 (0.02) | 0.03 (0.02) | 2.73 (0.37) |
| icp | 43.92 (16.85) | 13.7 (4.72) | 0.37 (0.2) | 0.41 (0.2) | 14.2 (5.34) | 0.05 (0.02) | 0.03 (0.02) | 2.68 (0.41) |
| mot | 61.57 (18.49) | 14.1 (5.53) | 0.26 (0.15) | 0.39 (0.2) | 14.91 (6.15) | 0.06 (0.02) | 0.03 (0.02) | 2.59 (0.46) |
| msc | 49.9 (18.29) | 13.47 (5) | 0.32 (0.21) | 0.37 (0.21) | 14.3 (5.44) | 0.06 (0.02) | 0.03 (0.02) | 2.62 (0.41) |
| rfp | 52.09 (9.12) | 9.96 (4.22) | 0.21 (0.19) | 0.24 (0.19) | 11.78 (4.91) | 0.06 (0.02) | 0.02 (0.02) | 2.35 (0.54) |
| tas | 85.91 (16.51) | 7.7 (5.6) | 0.1 (0.09) | 0.45 (0.27) | 6.96 (5.55) | 0.06 (0.02) | 0.01 (0.01) | 1.81 (0.86) |
| wpe | 38.08 (18.45) | 13.13 (5.42) | 0.42 (0.23) | 0.36 (0.2) | 14.2 (6.09) | 0.05 (0.02) | 0.03 (0.02) | 2.65 (0.42) |

### **S2** Group differences in average process characteristics

#### Mean

Anova results for group differences in the average process
characteristics per TPQ scale, with p-values and BH-corrected
q-values.

| TPQ scale | metric | F | p | q |
| --- | --- | --- | --- | --- |
| epi | mean | 2.682707 | 0.0517898 | 0.3275504 |
| icp | mean | 2.806648 | 0.0448577 | 0.3275504 |
| mot | mean | 3.655524 | 0.0158976 | 0.2967560 |
| msc | mean | 2.630999 | 0.0555758 | 0.3275504 |
| rfp | mean | 1.158296 | 0.3311241 | 0.6394121 |
| tas | mean | 1.470074 | 0.2292330 | 0.5564355 |
| wpe | mean | 2.389578 | 0.0745157 | 0.3397118 |

#### SD

Anova results for group differences in the average process
characteristics per TPQ scale, with p-values and BH-corrected
q-values.

| TPQ scale | metric | F | p | q |
| --- | --- | --- | --- | --- |
| epi | sd | 0.2825845 | 0.8378307 | 0.8687047 |
| icp | sd | 1.3431970 | 0.2657248 | 0.5952236 |
| mot | sd | 0.3558165 | 0.7850471 | 0.8687047 |
| msc | sd | 3.0403293 | 0.0330326 | 0.3275504 |
| rfp | sd | 0.8794018 | 0.4556152 | 0.7962664 |
| tas | sd | 2.6435799 | 0.0550059 | 0.3275504 |
| wpe | sd | 1.2396796 | 0.3005975 | 0.6011951 |

#### Rel. SD

Anova results for group differences in the average process
characteristics per TPQ scale, with p-values and BH-corrected
q-values.

| TPQ scale | metric | F | p | q |
| --- | --- | --- | --- | --- |
| epi | rel\_sd | 2.5746941 | 0.0584911 | 0.3275504 |
| icp | rel\_sd | 0.3575452 | 0.7838063 | 0.8687047 |
| mot | rel\_sd | 0.8528068 | 0.4692284 | 0.7962664 |
| msc | rel\_sd | 0.2611362 | 0.8531921 | 0.8687047 |
| rfp | rel\_sd | 1.4990335 | 0.2212945 | 0.5564355 |
| tas | rel\_sd | 2.4191229 | 0.0723126 | 0.3397118 |
| wpe | rel\_sd | 0.6334652 | 0.5956295 | 0.8687047 |

#### ACF

Anova results for group differences in the average process
characteristics per TPQ scale, with p-values and BH-corrected
q-values.

| TPQ scale | metric | F | p | q |
| --- | --- | --- | --- | --- |
| epi | acf1 | 1.7677920 | 0.1598464 | 0.5559903 |
| icp | acf1 | 1.5858907 | 0.1985680 | 0.5559903 |
| mot | acf1 | 2.3432472 | 0.0788617 | 0.3397118 |
| msc | acf1 | 1.4354332 | 0.2384724 | 0.5564355 |
| rfp | acf1 | 2.5958663 | 0.0575836 | 0.3275504 |
| tas | acf1 | 0.7764725 | 0.5105279 | 0.8408695 |
| wpe | acf1 | 0.9514077 | 0.4199628 | 0.7839306 |

#### RMSSD

Anova results for group differences in the average process
characteristics per TPQ scale, with p-values and BH-corrected
q-values.

| TPQ scale | metric | F | p | q |
| --- | --- | --- | --- | --- |
| epi | rmssd | 0.5896646 | 0.6235642 | 0.8687047 |
| icp | rmssd | 0.5509137 | 0.6489772 | 0.8687047 |
| mot | rmssd | 0.3182288 | 0.8121558 | 0.8687047 |
| msc | rmssd | 0.4551282 | 0.7143692 | 0.8687047 |
| rfp | rmssd | 1.2608426 | 0.2937012 | 0.6011951 |
| tas | rmssd | 4.3425988 | 0.0068956 | 0.1930760 |
| wpe | rmssd | 0.3256094 | 0.8068307 | 0.8687047 |

#### PAC

Anova results for group differences in the average process
characteristics per TPQ scale, with p-values and BH-corrected
q-values.

| TPQ scale | metric | F | p | q |
| --- | --- | --- | --- | --- |
| epi | pac | 1.2983733 | 0.2808846 | 0.6011951 |
| icp | pac | 0.4503651 | 0.7177373 | 0.8687047 |
| mot | pac | 0.3430960 | 0.7942176 | 0.8687047 |
| msc | pac | 0.4102050 | 0.7461100 | 0.8687047 |
| rfp | pac | 1.9886594 | 0.1216232 | 0.4864930 |
| tas | pac | 1.4416998 | 0.2365551 | 0.5564355 |
| wpe | pac | 1.6310270 | 0.1883988 | 0.5559903 |

#### Entropy

Anova results for group differences in the average process
characteristics per TPQ scale, with p-values and BH-corrected
q-values.

| TPQ scale | metric | F | p | q |
| --- | --- | --- | --- | --- |
| epi | shan | 1.6188012 | 0.1918268 | 0.5559903 |
| icp | shan | 0.2919447 | 0.8311004 | 0.8687047 |
| mot | shan | 0.5439038 | 0.6536319 | 0.8687047 |
| msc | shan | 0.8728449 | 0.4589268 | 0.7962664 |
| rfp | shan | 1.8405785 | 0.1461890 | 0.5457721 |
| tas | shan | 0.6506473 | 0.5848256 | 0.8687047 |
| wpe | shan | 0.1239097 | 0.9457207 | 0.9457207 |

#### DC

Anova results for group differences in the average process
characteristics per TPQ scale, with p-values and BH-corrected
q-values.

| TPQ scale | metric | F | p | q |
| --- | --- | --- | --- | --- |
| epi | dc | 0.6860878 | 0.5632912 | 0.8687047 |
| icp | dc | 0.4865426 | 0.6925860 | 0.8687047 |
| mot | dc | 0.4209012 | 0.7384999 | 0.8687047 |
| msc | dc | 0.4642297 | 0.7080339 | 0.8687047 |
| rfp | dc | 1.6610046 | 0.1826672 | 0.5559903 |
| tas | dc | 4.3955356 | 0.0065566 | 0.1930760 |
| wpe | dc | 0.4600577 | 0.7109699 | 0.8687047 |

### **S3** Changes over time in process characteristics

#### Mean

Average change over time for process characteristics per TPQ
scale (calculated in 14-day moving windows), given as the average slope
of the time trend from mixed models.

| TPQ scale | estimate | conf.low | conf.high | p.value | q |
| --- | --- | --- | --- | --- | --- |
| epi | -0.1058 | -0.1389 | -0.0727 | 0 | 0e+00 |
| icp | 0.1589 | 0.1281 | 0.1896 | 0 | 0e+00 |
| mot | 0.1147 | 0.0839 | 0.1455 | 0 | 0e+00 |
| msc | 0.1199 | 0.0874 | 0.1525 | 0 | 0e+00 |
| rfp | -0.0422 | -0.0613 | -0.0231 | 0 | 0e+00 |
| tas | 0.0530 | 0.0278 | 0.0781 | 0 | 1e-04 |
| wpe | 0.0815 | 0.0531 | 0.1099 | 0 | 0e+00 |

#### SD

Average change over time for process characteristics per TPQ
scale (calculated in 14-day moving windows), given as the average slope
of the time trend from mixed models.

| TPQ scale | estimate | conf.low | conf.high | p.value | q |
| --- | --- | --- | --- | --- | --- |
| epi | -0.0292 | -0.0418 | -0.0165 | 0 | 0 |
| icp | -0.0264 | -0.0374 | -0.0154 | 0 | 0 |
| mot | -0.0396 | -0.0530 | -0.0263 | 0 | 0 |
| msc | -0.0390 | -0.0524 | -0.0256 | 0 | 0 |
| rfp | -0.0595 | -0.0720 | -0.0469 | 0 | 0 |
| tas | -0.0309 | -0.0449 | -0.0168 | 0 | 0 |
| wpe | -0.0289 | -0.0416 | -0.0161 | 0 | 0 |

#### Rel. SD

Average change over time for process characteristics per TPQ
scale (calculated in 14-day moving windows), given as the average slope
of the time trend from mixed models.

| TPQ scale | estimate | conf.low | conf.high | p.value | q |
| --- | --- | --- | --- | --- | --- |
| epi | 0.0008 | 0.0002 | 0.0013 | 0.0038 | 0.0056 |
| icp | -0.0017 | -0.0021 | -0.0013 | 0.0000 | 0.0000 |
| mot | -0.0011 | -0.0015 | -0.0008 | 0.0000 | 0.0000 |
| msc | -0.0014 | -0.0019 | -0.0009 | 0.0000 | 0.0000 |
| rfp | -0.0009 | -0.0012 | -0.0005 | 0.0000 | 0.0000 |
| tas | -0.0004 | -0.0007 | -0.0002 | 0.0000 | 0.0001 |
| wpe | -0.0013 | -0.0017 | -0.0008 | 0.0000 | 0.0000 |

#### ACF

Average change over time for process characteristics per TPQ
scale (calculated in 14-day moving windows), given as the average slope
of the time trend from mixed models.

| TPQ scale | estimate | conf.low | conf.high | p.value | q |
| --- | --- | --- | --- | --- | --- |
| epi | 0.0012 | 5e-04 | 0.0019 | 0.0005 | 0.0008 |
| icp | 0.0006 | -1e-04 | 0.0012 | 0.0816 | 0.1023 |
| mot | 0.0006 | -1e-04 | 0.0012 | 0.0822 | 0.1023 |
| msc | 0.0002 | -4e-04 | 0.0009 | 0.4482 | 0.4827 |
| rfp | -0.0001 | -7e-04 | 0.0006 | 0.8512 | 0.8512 |
| tas | -0.0003 | -1e-03 | 0.0004 | 0.4003 | 0.4396 |
| wpe | 0.0009 | 3e-04 | 0.0016 | 0.0042 | 0.0058 |

#### RMSSD

Average change over time for process characteristics per TPQ
scale (calculated in 14-day moving windows), given as the average slope
of the time trend from mixed models.

| TPQ scale | estimate | conf.low | conf.high | p.value | q |
| --- | --- | --- | --- | --- | --- |
| epi | -0.0508 | -0.0675 | -0.0342 | 0 | 0e+00 |
| icp | -0.0368 | -0.0506 | -0.0230 | 0 | 0e+00 |
| mot | -0.0547 | -0.0704 | -0.0390 | 0 | 0e+00 |
| msc | -0.0520 | -0.0685 | -0.0355 | 0 | 0e+00 |
| rfp | -0.0774 | -0.0940 | -0.0607 | 0 | 0e+00 |
| tas | -0.0348 | -0.0512 | -0.0185 | 0 | 1e-04 |
| wpe | -0.0470 | -0.0626 | -0.0315 | 0 | 0e+00 |

#### PAC

Average change over time for process characteristics per TPQ
scale (calculated in 14-day moving windows), given as the average slope
of the time trend from mixed models.

| TPQ scale | estimate | conf.low | conf.high | p.value | q |
| --- | --- | --- | --- | --- | --- |
| epi | 1e-04 | 0e+00 | 2e-04 | 0.0903 | 0.1099 |
| icp | 1e-04 | 0e+00 | 2e-04 | 0.0040 | 0.0057 |
| mot | 1e-04 | 0e+00 | 2e-04 | 0.2382 | 0.2779 |
| msc | 1e-04 | 0e+00 | 2e-04 | 0.1263 | 0.1505 |
| rfp | 0e+00 | -1e-04 | 1e-04 | 0.4942 | 0.5221 |
| tas | 0e+00 | -1e-04 | 1e-04 | 0.6582 | 0.6702 |
| wpe | 1e-04 | 0e+00 | 2e-04 | 0.0055 | 0.0075 |

#### Entropy

Average change over time for process characteristics per TPQ
scale (calculated in 14-day moving windows), given as the average slope
of the time trend from mixed models.

| TPQ scale | estimate | conf.low | conf.high | p.value | q |
| --- | --- | --- | --- | --- | --- |
| epi | -0.0004 | -0.0012 | 3e-04 | 0.2695 | 0.3080 |
| icp | -0.0003 | -0.0010 | 3e-04 | 0.3182 | 0.3564 |
| mot | -0.0011 | -0.0019 | -4e-04 | 0.0036 | 0.0054 |
| msc | -0.0010 | -0.0018 | -2e-04 | 0.0149 | 0.0199 |
| rfp | -0.0017 | -0.0025 | -8e-04 | 0.0002 | 0.0003 |
| tas | -0.0015 | -0.0027 | -2e-04 | 0.0189 | 0.0246 |
| wpe | -0.0002 | -0.0009 | 5e-04 | 0.6067 | 0.6292 |

#### DC

Average change over time for process characteristics per TPQ
scale (calculated in 14-day moving windows), given as the average slope
of the time trend from mixed models.

| TPQ scale | estimate | conf.low | conf.high | p.value | q |
| --- | --- | --- | --- | --- | --- |
| epi | -2e-04 | -3e-04 | -1e-04 | 0e+00 | 0e+00 |
| icp | -1e-04 | -2e-04 | -1e-04 | 1e-04 | 1e-04 |
| mot | -2e-04 | -3e-04 | -1e-04 | 0e+00 | 0e+00 |
| msc | -2e-04 | -3e-04 | -1e-04 | 0e+00 | 0e+00 |
| rfp | -2e-04 | -3e-04 | -1e-04 | 0e+00 | 0e+00 |
| tas | -1e-04 | -1e-04 | 0e+00 | 1e-04 | 1e-04 |
| wpe | -2e-04 | -3e-04 | -1e-04 | 0e+00 | 0e+00 |

### **S4** Group differences in process characteristics’ changes over time

#### Mean

Anova results for group differences in the changes over time in
process characteristics, with p-values and BH-corrected
q-values.

| TPQ scale | F | p | q |
| --- | --- | --- | --- |
| epi | 1.6255474 | 0.190 | 0.634 |
| icp | 1.6057472 | 0.194 | 0.634 |
| mot | 0.8356488 | 0.478 | 0.986 |
| msc | 0.2459699 | 0.864 | 0.986 |
| rfp | 0.1562078 | 0.925 | 0.986 |
| tas | 0.5369229 | 0.658 | 0.986 |
| wpe | 1.9705019 | 0.125 | 0.634 |

#### SD

Anova results for group differences in the changes over time in
process characteristics, with p-values and BH-corrected
q-values.

| TPQ scale | F | p | q |
| --- | --- | --- | --- |
| epi | 0.9631017 | 0.414 | 0.986 |
| icp | 0.4467838 | 0.720 | 0.986 |
| mot | 1.7279912 | 0.168 | 0.634 |
| msc | 0.6711649 | 0.572 | 0.986 |
| rfp | 0.2936176 | 0.830 | 0.986 |
| tas | 1.6973207 | 0.174 | 0.634 |
| wpe | 0.4791246 | 0.698 | 0.986 |

#### Rel. SD

Anova results for group differences in the changes over time in
process characteristics, with p-values and BH-corrected
q-values.

| TPQ scale | F | p | q |
| --- | --- | --- | --- |
| epi | 2.3845825 | 0.075 | 0.634 |
| icp | 0.8037984 | 0.495 | 0.986 |
| mot | 2.5918884 | 0.058 | 0.634 |
| msc | 0.7374494 | 0.533 | 0.986 |
| rfp | 1.0454629 | 0.378 | 0.986 |
| tas | 1.2930157 | 0.283 | 0.834 |
| wpe | 1.6660424 | 0.181 | 0.634 |

#### ACF

Anova results for group differences in the changes over time in
process characteristics, with p-values and BH-corrected
q-values.

| TPQ scale | F | p | q |
| --- | --- | --- | --- |
| epi | 1.8798553 | 0.139 | 0.634 |
| icp | 1.0206555 | 0.388 | 0.986 |
| mot | 0.6682011 | 0.574 | 0.986 |
| msc | 0.1152195 | 0.951 | 0.986 |
| rfp | 0.1772853 | 0.912 | 0.986 |
| tas | 0.6051238 | 0.613 | 0.986 |
| wpe | 0.0145882 | 0.998 | 0.998 |

#### RMSSD

Anova results for group differences in the changes over time in
process characteristics, with p-values and BH-corrected
q-values.

| TPQ scale | F | p | q |
| --- | --- | --- | --- |
| epi | 0.4616813 | 0.710 | 0.986 |
| icp | 0.4475098 | 0.720 | 0.986 |
| mot | 1.9308322 | 0.131 | 0.634 |
| msc | 0.2483711 | 0.862 | 0.986 |
| rfp | 0.2403290 | 0.868 | 0.986 |
| tas | 1.5679719 | 0.204 | 0.634 |
| wpe | 0.1444801 | 0.933 | 0.986 |

#### PAC

Anova results for group differences in the changes over time in
process characteristics, with p-values and BH-corrected
q-values.

| TPQ scale | F | p | q |
| --- | --- | --- | --- |
| epi | 0.9495337 | 0.421 | 0.986 |
| icp | 2.5688465 | 0.060 | 0.634 |
| mot | 0.6933729 | 0.559 | 0.986 |
| msc | 2.1032780 | 0.106 | 0.634 |
| rfp | 1.9329002 | 0.130 | 0.634 |
| tas | 3.3604959 | 0.023 | 0.634 |
| wpe | 0.3650011 | 0.778 | 0.986 |

#### Entropy

Anova results for group differences in the changes over time in
process characteristics, with p-values and BH-corrected
q-values.

| TPQ scale | F | p | q |
| --- | --- | --- | --- |
| epi | 0.8199552 | 0.487 | 0.986 |
| icp | 0.5735307 | 0.634 | 0.986 |
| mot | 0.1231272 | 0.946 | 0.986 |
| msc | 3.4797060 | 0.019 | 0.634 |
| rfp | 2.9943920 | 0.036 | 0.634 |
| tas | 0.3829409 | 0.766 | 0.986 |
| wpe | 0.5152224 | 0.673 | 0.986 |

#### DC

Anova results for group differences in the changes over time in
process characteristics, with p-values and BH-corrected
q-values.

| TPQ scale | F | p | q |
| --- | --- | --- | --- |
| epi | 0.0422809 | 0.988 | 0.998 |
| icp | 0.1898957 | 0.903 | 0.986 |
| mot | 2.0931613 | 0.108 | 0.634 |
| msc | 0.3875818 | 0.762 | 0.986 |
| rfp | 0.6784945 | 0.568 | 0.986 |
| tas | 0.3496506 | 0.789 | 0.986 |
| wpe | 0.1621794 | 0.922 | 0.986 |

### **S5** Prediction of improvement by changes over time in process characteristics

#### Mean

Logistic regression results from process characteristics
predicting clinical improvement, with p-values and BH-corrected
q-values.

| dyn | TPQ scale | OR | p | q |
| --- | --- | --- | --- | --- |
| mean | wpe | 2.0494692 | 0.0000057 | 0.0002735 |
| mean | msc | 1.8568383 | 0.0000743 | 0.0017825 |
| mean | icp | 1.6837218 | 0.0004408 | 0.0061861 |
| mean | mot | 1.4830147 | 0.0075606 | 0.0403232 |
| mean | tas | 1.1356105 | 0.3664536 | 0.5173462 |
| mean | rfp | 0.9024192 | 0.4656832 | 0.6386513 |

#### SD

Logistic regression results from process characteristics
predicting clinical improvement, with p-values and BH-corrected
q-values.

| dyn | TPQ scale | OR | p | q |
| --- | --- | --- | --- | --- |
| sd | rfp | 0.6654981 | 0.0038852 | 0.0266411 |
| sd | tas | 0.7451243 | 0.0586908 | 0.1760723 |
| sd | mot | 0.8088990 | 0.1302059 | 0.2659688 |
| sd | msc | 0.8170398 | 0.1468278 | 0.2819093 |
| sd | wpe | 0.8369883 | 0.1918116 | 0.3541137 |
| sd | icp | 0.8760039 | 0.3387902 | 0.5081853 |

#### Rel. SD

Logistic regression results from process characteristics
predicting clinical improvement, with p-values and BH-corrected
q-values.

| dyn | TPQ scale | OR | p | q |
| --- | --- | --- | --- | --- |
| rel\_sd | msc | 0.5572825 | 0.0006444 | 0.0061861 |
| rel\_sd | wpe | 0.5827266 | 0.0005561 | 0.0061861 |
| rel\_sd | icp | 0.6538813 | 0.0031126 | 0.0249011 |
| rel\_sd | mot | 0.6697998 | 0.0070056 | 0.0403232 |
| rel\_sd | rfp | 0.7279501 | 0.0511141 | 0.1635650 |
| rel\_sd | tas | 0.7869438 | 0.0938779 | 0.2186072 |

#### ACF

Logistic regression results from process characteristics
predicting clinical improvement, with p-values and BH-corrected
q-values.

| dyn | TPQ scale | OR | p | q |
| --- | --- | --- | --- | --- |
| acf1 | rfp | 0.7405665 | 0.0377196 | 0.1442954 |
| acf1 | icp | 0.8022581 | 0.1089690 | 0.2377506 |
| acf1 | msc | 0.9158511 | 0.5301039 | 0.7068052 |
| acf1 | tas | 1.1103204 | 0.5505955 | 0.7142861 |
| acf1 | wpe | 0.9411574 | 0.6652354 | 0.7982825 |
| acf1 | mot | 0.9546506 | 0.7398585 | 0.8553831 |

#### RMSSD

Logistic regression results from process characteristics
predicting clinical improvement, with p-values and BH-corrected
q-values.

| dyn | TPQ scale | OR | p | q |
| --- | --- | --- | --- | --- |
| rmssd | rfp | 0.7472910 | 0.0326529 | 0.1442954 |
| rmssd | tas | 0.7421653 | 0.0486165 | 0.1635650 |
| rmssd | wpe | 0.8382859 | 0.2017411 | 0.3586508 |
| rmssd | msc | 0.8534222 | 0.2482929 | 0.4256449 |
| rmssd | mot | 0.8556100 | 0.2647429 | 0.4381951 |
| rmssd | icp | 1.0045051 | 0.9744172 | 0.9885166 |

#### PAC

Logistic regression results from process characteristics
predicting clinical improvement, with p-values and BH-corrected
q-values.

| dyn | TPQ scale | OR | p | q |
| --- | --- | --- | --- | --- |
| pac | tas | 0.8792105 | 0.3601350 | 0.5173462 |
| pac | rfp | 1.0787861 | 0.5864195 | 0.7407404 |
| pac | msc | 0.9341279 | 0.6276005 | 0.7724314 |
| pac | mot | 0.9562633 | 0.7484602 | 0.8553831 |
| pac | wpe | 1.0384542 | 0.7876379 | 0.8607449 |
| pac | icp | 1.0218308 | 0.8777883 | 0.9341769 |

#### Entropy

Logistic regression results from process characteristics
predicting clinical improvement, with p-values and BH-corrected
q-values.

| dyn | TPQ scale | OR | p | q |
| --- | --- | --- | --- | --- |
| shan | rfp | 0.7829538 | 0.0956407 | 0.2186072 |
| shan | tas | 1.2759038 | 0.0815992 | 0.2186072 |
| shan | msc | 0.8107099 | 0.1329844 | 0.2659688 |
| shan | mot | 0.8589632 | 0.2810046 | 0.4496073 |
| shan | icp | 0.8656818 | 0.3063349 | 0.4743250 |
| shan | wpe | 0.9979858 | 0.9885166 | 0.9885166 |

#### DC

Logistic regression results from process characteristics
predicting clinical improvement, with p-values and BH-corrected
q-values.

| dyn | TPQ scale | OR | p | q |
| --- | --- | --- | --- | --- |
| dc | rfp | 0.7471427 | 0.0376475 | 0.1442954 |
| dc | wpe | 0.7463972 | 0.0390800 | 0.1442954 |
| dc | msc | 0.7884236 | 0.0930007 | 0.2186072 |
| dc | tas | 0.7944156 | 0.0888518 | 0.2186072 |
| dc | icp | 0.9633774 | 0.7890162 | 0.8607449 |
| dc | mot | 0.9817393 | 0.8952529 | 0.9341769 |

### **S6** Group differences in the association of improvement and changes over time

#### Mean

Likelihood-ratio tests for the diagnosis × trend interaction
(full vs. reduced model).

| dyn | TPQ scale | Chisq | p | q |
| --- | --- | --- | --- | --- |
| mean | icp | 3.1950991 | 0.3625118 | 0.7901905 |
| mean | mot | 1.9198394 | 0.5892099 | 0.8838148 |
| mean | msc | 2.3597337 | 0.5011742 | 0.8416258 |
| mean | rfp | 3.2506432 | 0.3545715 | 0.7901905 |
| mean | tas | 0.2490449 | 0.9693084 | 0.9716887 |
| mean | wpe | 3.8877296 | 0.2738453 | 0.7901905 |

#### SD

Likelihood-ratio tests for the diagnosis × trend interaction
(full vs. reduced model).

| dyn | TPQ scale | Chisq | p | q |
| --- | --- | --- | --- | --- |
| sd | icp | 0.8928673 | 0.8271487 | 0.9626801 |
| sd | mot | 4.8591264 | 0.1824081 | 0.7901905 |
| sd | msc | 5.6841913 | 0.1280277 | 0.7901905 |
| sd | rfp | 1.4626462 | 0.6909180 | 0.9475447 |
| sd | tas | 5.4578314 | 0.1411826 | 0.7901905 |
| sd | wpe | 9.4118526 | 0.0242878 | 0.7901905 |

#### Rel. SD

Likelihood-ratio tests for the diagnosis × trend interaction
(full vs. reduced model).

| dyn | TPQ scale | Chisq | p | q |
| --- | --- | --- | --- | --- |
| rel\_sd | icp | 0.7525480 | 0.8607799 | 0.9626801 |
| rel\_sd | mot | 0.2812289 | 0.9635193 | 0.9716887 |
| rel\_sd | msc | 1.6873730 | 0.6397447 | 0.9031690 |
| rel\_sd | rfp | 2.8734093 | 0.4115576 | 0.7901905 |
| rel\_sd | tas | 5.5042991 | 0.1383817 | 0.7901905 |
| rel\_sd | wpe | 3.7178018 | 0.2935930 | 0.7901905 |

#### ACF

Likelihood-ratio tests for the diagnosis × trend interaction
(full vs. reduced model).

| dyn | TPQ scale | Chisq | p | q |
| --- | --- | --- | --- | --- |
| acf1 | icp | 2.003038 | 0.5717763 | 0.8838148 |
| acf1 | mot | 3.923923 | 0.2697971 | 0.7901905 |
| acf1 | msc | 2.338405 | 0.5052035 | 0.8416258 |
| acf1 | rfp | 2.885529 | 0.4096132 | 0.7901905 |
| acf1 | tas | 4.935450 | 0.1765853 | 0.7901905 |
| acf1 | wpe | 5.410840 | 0.1440697 | 0.7901905 |

#### RMSSD

Likelihood-ratio tests for the diagnosis × trend interaction
(full vs. reduced model).

| dyn | TPQ scale | Chisq | p | q |
| --- | --- | --- | --- | --- |
| rmssd | icp | 0.2353599 | 0.9716887 | 0.9716887 |
| rmssd | mot | 1.9452172 | 0.5838546 | 0.8838148 |
| rmssd | msc | 3.2940713 | 0.3484687 | 0.7901905 |
| rmssd | rfp | 2.3211445 | 0.5084823 | 0.8416258 |
| rmssd | tas | 4.4298859 | 0.2186302 | 0.7901905 |
| rmssd | wpe | 3.0553628 | 0.3831678 | 0.7901905 |

#### PAC

Likelihood-ratio tests for the diagnosis × trend interaction
(full vs. reduced model).

| dyn | TPQ scale | Chisq | p | q |
| --- | --- | --- | --- | --- |
| pac | icp | 4.9332011 | 0.1767543 | 0.7901905 |
| pac | mot | 1.7808327 | 0.6191148 | 0.9005306 |
| pac | msc | 6.1781906 | 0.1032555 | 0.7901905 |
| pac | rfp | 1.1935881 | 0.7545426 | 0.9531064 |
| pac | tas | 7.3813193 | 0.0606876 | 0.7901905 |
| pac | wpe | 0.4169313 | 0.9367231 | 0.9716887 |

#### Enropy

Likelihood-ratio tests for the diagnosis × trend interaction
(full vs. reduced model).

| dyn | TPQ scale | Chisq | p | q |
| --- | --- | --- | --- | --- |
| shan | icp | 3.8472159 | 0.2784417 | 0.7901905 |
| shan | mot | 2.6543560 | 0.4480398 | 0.8271504 |
| shan | msc | 1.1081937 | 0.7750965 | 0.9539649 |
| shan | rfp | 0.7457206 | 0.8624009 | 0.9626801 |
| shan | tas | 1.2397546 | 0.7434861 | 0.9531064 |
| shan | wpe | 1.2891869 | 0.7317024 | 0.9531064 |

#### DC

Likelihood-ratio tests for the diagnosis × trend interaction
(full vs. reduced model).

| dyn | TPQ scale | Chisq | p | q |
| --- | --- | --- | --- | --- |
| dc | icp | 0.5515072 | 0.9074382 | 0.9716887 |
| dc | mot | 0.9391914 | 0.8159612 | 0.9626801 |
| dc | msc | 7.6300570 | 0.0543092 | 0.7901905 |
| dc | rfp | 3.0224910 | 0.3881705 | 0.7901905 |
| dc | tas | 4.0325251 | 0.2579734 | 0.7901905 |
| dc | wpe | 3.3050292 | 0.3469433 | 0.7901905 |
